# Training the Trainer: Faculty From Across Multiple Specialties Show Improved Confidence, Knowledge and Skill in Point of Care Ultrasound After a Short Intervention

**DOI:** 10.7759/cureus.11821

**Published:** 2020-12-01

**Authors:** Frances M Russell, Audrey Herbert, Bita Zakeri, Mary Blaha, Robinson M Ferre, Elisa J Sarmiento, Paul M Wallach

**Affiliations:** 1 Emergency Medicine, Indiana University, Indianapolis, USA; 2 Education and Continuing Medical Education, Indiana University, Indianapolis, USA; 3 Emergency Medicine, Indiana University School of Medicine, Indianapolis, USA; 4 Department of Internal Medicine, Office of the Dean, Indiana University, Indianapolis, USA

**Keywords:** point-of-care-ultrasound, undergraduate medical education, pre-post study

## Abstract

Objectives

Lack of faculty skill and confidence in performing and teaching point-of-care ultrasound (POCUS) remains a significant barrier to implementation of a longitudinal ultrasound curriculum in undergraduate medical education. Our objective was to assess faculty comfort, knowledge and skill with performing and teaching POCUS before and after a focused workshop.

Methods

This was a prospective study assessing faculty from multiple specialties. Faculty completed a pre- and post-workshop survey and ultrasound knowledge assessment, and a post-workshop objective structured clinical examination (OSCE) to assess ability to perform POCUS. Differences between pre- and post-workshop responses were analyzed using Fisher's Exact and Wilcoxon tests, and statistical significance was accepted for p<0.05.

Results

We analyzed data on 78 faculty from multiple disciplines. Faculty had a median of 7.5 years of experience with medical student teaching. Sixty-eight percent of faculty had performed <25 prior ultrasound (US) examinations. Comparing pre- to post-workshop responses, we found significant reductions in barriers to using US, and improved confidence with using, obtaining and interpreting POCUS (p<0.01). Faculty felt significantly more comfortable with the idea of teaching medical students POCUS (p<0.01). POCUS knowledge improved from 50% to 86% (p<0.01). On the post-workshop OSCE, 90% of anatomic structures were correctly identified with a median image quality of 4 out of 5.

Conclusion

After attending a six-hour workshop, faculty across multiple specialties had increased confidence with using and teaching POCUS, showed improved knowledge, and were able to correctly identify pertinent anatomic structures with ultrasound while obtaining good image quality.

## Introduction

Point-of-care ultrasound (POCUS) is an important bedside tool that clinicians use to quickly diagnose and guide treatment decisions in both the inpatient and outpatient clinical setting. In many clinical settings, it has become the standard of care for guiding procedures, screening for and diagnosing a variety of conditions [1]. In response to the growing use of POCUS by an array of clinical specialties, many medical schools have implemented POCUS curricula over the past decade [2-4]. Implementation requires local faculty knowledge and expertise to help with designing and executing a POCUS curriculum. Implementation of such a program is typically led by faculty from specialties that use ultrasound (US) frequently in their practice, such as emergency medicine physicians, intensivists, obstetricians, radiologists, and cardiologists [3].

While POCUS has been increasingly incorporated into some aspects of the undergraduate medical education (UME) curriculum, only a limited number of medical schools have a longitudinal four-year integrated POCUS curriculum [3,4]. This is due, in part, to the many obstacles in creating and implementing such a curriculum [3,5,6]. One such factor, lack of skilled faculty instructors [5], remains an important barrier for many institutions [3,6]. Training faculty through POCUS instruction may help meet this need. 

While a majority of the literature surrounding ultrasound education focuses on medical students and residents, there remains limited data assessing faculty POCUS training. Prior studies [7-9] focused on faculty ultrasound education and training are limited by small sample sizes, focus on a specific exam only, or are limited to a specific specialty. 

To meet the needs of implementing a longitudinal POCUS curriculum at our institution, we developed a variety of six-hour workshops that used a combination of lecture and hands-on instruction to grow the number of physician and non-physician faculty who could assist with UME POCUS instruction. As part of that instruction, we gathered pre and post data to assess the effectiveness of a six-hour POCUS workshop. The purpose of this study was to assess confidence regarding POCUS use, knowledge related to POCUS exams, and the ability to obtain interpretable POCUS images among faculty of various specialty backgrounds after a six-hour POCUS workshop.

## Materials and methods

Study Design

This was a prospective, cross-sectional study that assessed confidence, knowledge and skill with POCUS, among faculty from multiple disciplines across the school of medicine. Faculty completed a pre- and post-workshop survey and knowledge assessment, and a post-workshop objective structured clinical examination (OSCE). Data were collected from February 2019 to January 2020. This study was deemed exempt by the Institutional Review Board with waiver of informed consent.

Surveys and Knowledge Assessments

The pre-workshop survey consisted of six questions and assessed specialty, years teaching medical students, and baseline ultrasound experience. We also evaluated barriers to POCUS utilization, and a 5-point Likert scale (1: strongly disagree to 5: strongly agree) was used to evaluate faculty’s confidence in using an ultrasound machine, obtaining and interpreting POCUS images, and confidence with teaching POCUS to medical students. 

The post-workshop survey consisted of two questions focused on barriers to POCUS use and confidence with POCUS, which mirrored the pre-workshop survey to allow for direct comparison.

Faculty completed pre- and post-workshop knowledge assessments composed of 10 multiple-choice questions. The pre-test assessed baseline POCUS knowledge pertinent to the modalities being taught. Questions covered indications for performing ultrasound, acquisition, interpretation, troubleshooting difficulties, and pathology.

POCUS Workshop

After completion of the pre-survey and knowledge assessment, faculty completed a single six-hour ultrasound training workshop. Faculty were recruited throughout the year via school-wide emails and newsletters to attend a POCUS workshop. No single faculty attended more than one workshop. Five workshops were held throughout the year and covered varied content. Topics taught included cardiac, focused assessment in trauma, vascular access, gallbladder, aorta and first-trimester obstetric ultrasound. Content was taught through in-person lectures and hands-on ultrasound training with standardized patients. Four 20-minute lectures were given during each workshop; four hours were dedicated to experiential learning. Hands-on scanning stations were limited to four participants per instructor, and used standardized patient volunteers for scanning normal anatomy. During hands-on instruction, instructors were able to give participants real-time feedback regarding ways to improve image acquisition. The workshop was taught by ultrasound trained emergency medicine faculty, emergency medicine ultrasound fellows, ultrasound trained internal medicine faculty, sonography technology students and a senior emergency medicine resident. 

Objective Structured Clinical Examination (OSCE)

One of three ultrasound trained faculty administered a single station OSCE after the post test and survey were completed. This was done in a one-on-one session without feedback and was blinded to the other faculty participants. Faculty were asked to acquire three specific pre-determined images and identify anatomic structures on ultrasound. For example, they were asked to identify a parasternal long axis view of the heart and identify the left ventricle.

We assessed hands-on skill for acquiring images on standardized patients. A 5-point Likert scale was used to assess the quality of images obtained as determined by ultrasound trained faculty at the bedside. The scale was defined as 1=unable to obtain image, 2=partial view, but inadequate for interpretation, 3=partial view, adequate for interpretation, 4=full view of structure of with minor adjustments needed, 5=excellent view.

Data Analysis

Differences between pre- and post-survey responses were analyzed using Fisher's Exact and Wilcoxon tests. A 5% significance level was used for all tests. We performed all statistical analyses using SAS version 9.4 (SAS Institute, Cary, NC).

## Results

Seventy-eight faculty completed one of five workshops held over the course of one year. Faculty disciplines included Internal Medicine (22%), Anesthesia (18%), Family Medicine (13%), Anatomy (10%), Emergency Medicine (7%), Surgery (5%) and other (3%). Faculty had a median of 7.5 years of experience with medical student bedside teaching. Thirty-seven percent of faculty (29) had no to very little prior experience using ultrasound, and another 22% (17) had some prior experience (Table 1). Thirty-six percent of faculty (28) had never performed an ultrasound previously, and 68% (53) had performed fewer than 25 ultrasound exams prior to the workshop.

**Table 1:** Table of faculty participant characteristics.

Faculty characteristics, n=78	
Specialty, n (%)	
Anatomy	10 (12.8)
Anesthesia	18 (23.1)
Emergency Medicine	7 (9.0)
Internal Medicine	22 (28.2)
Family Medicine	13 (16.7)
Surgery	5 (6.4)
Other	3 (3.8)
Years teaching students, Median (Min-Max)	7.5 (0.0-35.0)
Prior ultrasound use	
None to very little	29 (37.2)
Some	17 (21.8)
Moderate	13 (16.7)
Large	13 (16.7)
Number of ultrasounds previously performed	
0	28 (35.9)
1-10	17 (21.8)
11-25	8 (10.2)
26-50	6 (7.7)
51-100	7 (9.0)
>100	8 (10.3)

When comparing barriers to ultrasound utilization pre- and post-workshop we found a significant reduction in most barriers (p<0.05) (Table 2). The main barriers identified pre-workshop were using an ultrasound machine, acquiring images, and interpreting images. These barriers, in addition to perceived personal skill level, were all significantly reduced post-workshop. Fourteen percent of faculty pre-workshop and 9% post-workshop felt that POCUS took too much time to perform (p=0.45). More faculty identified as having “no barriers” to ultrasound use post-workshop, although this was not statistically significant. 

**Table 2:** Barriers to ultrasound use Table of barriers to point-of-care ultrasound (POCUS) use pre- and post workshop. Participants selected any barrier (from a list) that applied to their personal use of POCUS.

Barriers to ultrasound use, n (%)			
	Pre Survey	Post Survey	P-Value
Don’t know how to use machine	37 (47.4)	2 (2.6)	0.0001
Don’t know how to acquire images	37 (47.4)	6 (7.7)	0.0001
Don’t know how to interpret images	42 (53.8)	8 (10.3)	0.0001
Comfort level of personal skills	52 (66.7)	31 (39.7)	0.0013
Don’t see utility of using it clinically	1 (1.3)	0 (0.0)	1.00
Takes too much to perform	11 (14.1)	7 (8.9)	0.4530
None	12 (15.4)	17 (21.8)	0.4107

Confidence in using, obtaining, and interpreting POCUS improved from pre- to post-workshop, as did confidence in teaching medical students POCUS (p<0.01) (Table 3). 

**Table 3:** Confidence Table of confidence with point-of-care ultrasound (POCUS) pre- and post workshop. US=Ultrasound.

Confidence, Median (Min-Max)			
	Pre Survey	Post Survey	P-Value
I feel confident using the US machine	3.0 (1.0-5.0)	4.0 (1.0-5.0)	0.0001
I feel confident obtaining US images	2.0 (1.0-5.0)	4.0 (1.0-5.0)	0.0001
I feel confident interpreting US images	2.0 (1.0-5.0)	4.0 (2.0-5.0)	0.0001
I feel confident teaching US to students	2.0 (1.0-5.0)	4.0 (1.0-5.0)	0.0001

Seventy-five out of 78 (96%) faculty completed both a pre- and post-workshop POCUS knowledge assessment. We found that scores significantly improved from pre- to post-workshop, 50% vs 85.7% (p<0.01) (Table 4). Scores significantly improved across all modalities, except gallbladder where both the pre- and post-workshop scores were high.

**Table 4:** Knowledge Assessment Scores Table of point-of-care ultrasound (POCUS) knowledge assessment pre- and post-workshop. Seventy-five faculty completed a pre- and post-knowledge assessment. Average overall score is listed pre and post. n = the number of questions answered related to that modality i.e. there were 534 responses for cardiac POCUS questions with corresponding percent correct pre and post.

Knowledge Assessment Scores, n=75			
	Pre Quiz	Post Quiz	P-Value
Total Score, range	50.0 (0.0-100.0)	85.7 (50.0-100.0)	0.0001
Cardiac, n=534	56.7	85.7	0.0001
Vascular access, n=291	57.3	81.1	0.0001
E-FAST, n=274	43.8	72.3	0.0001
Aorta, n=168	42.9	82.1	0.0001
Gallbladder, n=32	75.0	91.7	0.2053
Obstetric, n=11	0.0	66.7	0.0455

Forty-four out of 78 (56%) faculty completed a post-workshop OSCE. Ninety percent of the anatomic structures imaged were correctly acquired on POCUS with a median image quality of 4 out of 5. See Table 5 for a breakdown of structures, percent accurately identified and median image quality. 

**Table 5:** Post-course hands-on assessment Table of post-course hands-on assessment. Forty-four faculty completed a post-course objective structured clinical examination. Number of anatomic structures imaged correctly are listed with percents. n = the number of images acquired for a specific view i.e. there were 44 faculty who imaged Morison’s pouch.

Post-course hands-on assessment, n=44		
Anatomic View, n	Number Correct n (%)	Median Image Quality (Min-Max)
Total	168 (89.8)	4.0 (1.0-5.0)
Proximal aorta short, n=35	31 (88.6)	4.0 (3.0-5.0)
Morison's pouch, n=44	35 (79.5)	4.0 (1.0-5.0)
Parasternal long axis view, n=44	41 (93.2)	4.0 (1.0-5.0)
Identify LV in parasternal view, n=44	41 (93.2)	4.0 (1.0-5.0)
Brachial vein, n=14	14 (100.0)	4.0 (1.0-50)
Internal jugular vein, n=6	6 (100.0)	4.0 (1.0-5.0)

## Discussion

Implementation of a longitudinal POCUS curriculum in UME requires having multiple trained instructors who are confident, skilled and knowledgeable [3,5,6]. The primary aim of this study was to assess faculty confidence, knowledge and skill with POCUS before and after a workshop. In this study we found that after a short six-hour workshop, in mainly faculty who were novice to ultrasound from varied specialties, barriers to ultrasound significantly reduced, and confidence with using the machine, obtaining images, and interpreting images significantly increased. Faculty felt significantly more confident teaching POCUS to medical students. They had significantly higher knowledge assessment scores (p<0.0001) and were able to correctly acquire images of anatomic structures 90% of the time with good image quality. 

This study was novel in that we assessed nearly 80 faculty, and included faculty from varied disciplines including both physicians and PhD anatomists. In addition, we assessed multiple POCUS examinations and not just a single modality. When looking at prior data on ultrasound education and training a majority focuses on medical students and residents, and very little addresses faculty training. This is the first and largest study to our knowledge to assess faculty from across an entire school of medicine before and after a POCUS training course. Prior studies have been small, focus on a specific exam only, or are limited to a specific specialty. Conlon et al. [7] retrospectively assessed the feasibility of an ultrasound training program implemented for pediatric critical care faculty and fellows after a two-day introductory course. They found the course to be feasible, but did not compare knowledge or skill from pre- to post training. The study was retrospective and small assessing 25 participants. Chisholm et al. [8] evaluated 14 emergency physician faculty members’ ability to perform cardiac POCUS after 12 hours of training. They found that 90% of faculty passed the knowledge assessment, and 85% of faculty achieved acceptable images during the post-course skills assessment. Lewiss et al. [9] studied 31 faculty who completed a nine-month training curriculum on aorta and pelvic ultrasound. They found that 67% of faculty completed pre-set requirements. These studies differ from ours in that they were smaller, and only assessed emergency medicine faculty. 

While training faculty is important for undergraduate ultrasound medical education, it is imperative to acknowledge that there are alternative options to faculty for medical students to receive POCUS training including peer to peer teaching, and ultrasound technology students [5]. 

Our data supports that a short POCUS workshop is effective in training faculty to identify anatomic structures and acquire important POCUS knowledge. Thus, the results of this study may be used to inform future decisions on POCUS curriculum design and implementation strategies in undergraduate medical education. 

Limitations

There were several limitations to this study. Workshops were voluntary creating selection bias. Faculty participants who attended the workshops consisted of two main categories: those interested in learning more about POCUS, and those reluctant and uneasy about using POCUS and skeptical that the trainings would provide them enough knowledge and skill to be able to teach POCUS to medical students. Thus, there was a large gap in interest of the participants within the workshops. Content between workshops varied and was based on POCUS implementation needs within different areas of the undergraduate medical education curriculum. Because of this participants did not learn the same content and did not take the same assessments as questions and hands-on assessments were designed to address each particular workshop. However, there was overlap in content taught between the different workshops. Also, the pre- and post-workshop surveys remained the same. Lastly, only 56% of faculty completed the post-workshop OSCE as it was not performed during the first workshop, faculty left early from other workshops or were missed. Hence data from the skills assessment is small and not representative of all faculty.

Future studies assessing faculty POCUS training should attempt to evaluate the same content for all participants, and assess all learners in knowledge and skill after training completion.

## Conclusions

In conclusion, faculty from multiple specialties were significantly more confident with using and teaching POCUS, showed improved POCUS knowledge, and were able to correctly identify pertinent anatomic structures with US while obtaining good image quality after a six-hour workshop. This data supports that a short workshop is effective in improving faculty confidence, knowledge and skill in POCUS.

## References

[1] Moore CL, Copel JA (2011). Point-of-care ultrasonography. N Engl J Med.

[2] Bahner DP, Goldman E, Way D, Royall NA, Liu YT (2014). The state of ultrasound education in U.S. medical schools: results of a national survey. Acad Med.

[3] Hoppmann RA, Rao VV, Poston MB (2010). An integrated ultrasound curriculum (iUSC) for medical students: 4-year experience. Crit Ultrasound J.

[4] Bahner DP, Adkins EJ, Hughes D, Barrie M, Bougler CT, Royall NA (2013). Integrated medical school ultrasound: development of an ultrasound vertical curriculum. Crit Ultrasound J.

[5] Ahn JS, French AJ, Thiessen ME, Kendall JL (2014). Training peer instructors for a combined ultrasound/physical exam curriculum. Tech Learn Med.

[6] Dinh VA, Fu J, Lu S, Chiem A, Fox J, Blaivas M (2016). Integration of ultrasound in medical education at united states medical schools: A national survey of directors' experiences. J Ultrasound Med.

[7] Conlon TW, Himebauch AS, Fitzgerald JC (2015). Implementation of a pediatric critical care focused bedside ultrasound training program in a large academic PICU. Pediatr Crit Care Med.

[8] Chisholm CB, Dodge WR, Balise RR, Williams SR, Gharahbaghian L, Beraud AS (2013). Focused cardiac ultrasound training: how much is enough?. J Emerg Med.

[9] Lewiss RE, Saul T, Del Rios M Acquiring credentials in bedside ultrasound: a cross-sectional survey. BMJ Open.

